# Photosynthesis

**DOI:** 10.1042/EBC20160016

**Published:** 2016-10-26

**Authors:** Matthew P. Johnson

**Affiliations:** Department of Molecular Biology and Biotechnology, University of Sheffield, Firth Court, Western Bank, Sheffield S10 2TN, U.K.

**Keywords:** membrane, photosynthesis, thylakoid

## Abstract

Photosynthesis sustains virtually all life on planet Earth providing the oxygen we breathe and the food we eat; it forms the basis of global food chains and meets the majority of humankind's current energy needs through fossilized photosynthetic fuels. The process of photosynthesis in plants is based on two reactions that are carried out by separate parts of the chloroplast. The light reactions occur in the chloroplast thylakoid membrane and involve the splitting of water into oxygen, protons and electrons. The protons and electrons are then transferred through the thylakoid membrane to create the energy storage molecules adenosine triphosphate (ATP) and nicotinomide–adenine dinucleotide phosphate (NADPH). The ATP and NADPH are then utilized by the enzymes of the Calvin–Benson cycle (the dark reactions), which converts CO_2_ into carbohydrate in the chloroplast stroma. The basic principles of solar energy capture, energy, electron and proton transfer and the biochemical basis of carbon fixation are explained and their significance is discussed.

## An overview of photosynthesis

### Introduction

Photosynthesis is the ultimate source of all of humankind's food and oxygen, whereas fossilized photosynthetic fuels provide ∼87% of the world's energy. It is the biochemical process that sustains the biosphere as the basis for the food chain. The oxygen produced as a by-product of photosynthesis allowed the formation of the ozone layer, the evolution of aerobic respiration and thus complex multicellular life.

Oxygenic photosynthesis involves the conversion of water and CO_2_ into complex organic molecules such as carbohydrates and oxygen. Photosynthesis may be split into the ‘light’ and ‘dark’ reactions. In the light reactions, water is split using light into oxygen, protons and electrons, and in the dark reactions, the protons and electrons are used to reduce CO_2_ to carbohydrate (given here by the general formula CH_2_O). The two processes can be summarized thus:

Light reactions:

$$2H_{2}O+\mathrm{light}\to O_{2}+4H^{+}+4e^{-}\left(\Delta G^{\circ }=+317\;\mathrm{kJ}\cdot {\mathrm{mol}}^{-1}\right)$$

Dark reactions:

$$CO_{2}+4H^{+}+4e^{-}\to CH_{2}O+H_{2}O\left(\Delta G^{\circ }=+162\;\mathrm{kJ}\cdot {\mathrm{mol}}^{-1}\right)$$

Overall:

$$H_{2}O+\mathrm{light}+CO_{2}\to CH_{2}O+O_{2}\left(\Delta G^{\circ }=+479\;\mathrm{kJ}\cdot {\mathrm{mol}}^{-1}\right)$$

The positive sign of the **standard free energy change of the reaction (Δ*G*°)** given above means that the reaction requires energy (**an endergonic reaction**). The energy required is provided by absorbed solar energy, which is converted into the chemical bond energy of the products (Box 1).

Box 1. **Standard free energy change**

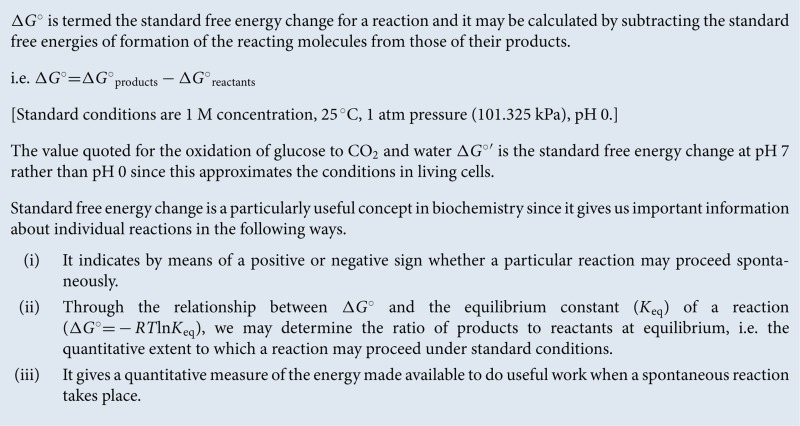

Photosynthesis converts ∼200 billion tonnes of CO_2_ into complex organic compounds annually and produces ∼140 billion tonnes of oxygen into the atmosphere. By facilitating conversion of solar energy into chemical energy, photosynthesis acts as the primary energy input into the global food chain. Nearly all living organisms use the complex organic compounds derived from photosynthesis as a source of energy. The breakdown of these organic compounds occurs via the process of aerobic respiration, which of course also requires the oxygen produced by photosynthesis.

$$CH_{2}O+O_{2}\to CO_{2}+H_{2}O\left(\Delta G^{\circ }=-479\;\mathrm{kJ}\cdot {\mathrm{mol}}^{-1}\right)$$

Unlike photosynthesis, aerobic respiration is an **exergonic process** (negative Δ*G*°) with the energy released being used by the organism to power biosynthetic processes that allow growth and renewal, mechanical work (such as muscle contraction or flagella rotation) and facilitating changes in chemical concentrations within the cell (e.g. accumulation of nutrients and expulsion of waste). The use of exergonic reactions to power endergonic ones associated with biosynthesis and housekeeping in biological organisms such that the overall free energy change is negative is known as ‘**coupling’.**

Photosynthesis and respiration are thus seemingly the reverse of one another, with the important caveat that both oxygen formation during photosynthesis and its utilization during respiration result in its liberation or incorporation respectively into water rather than CO_2_. In addition, glucose is one of several possible products of photosynthesis with amino acids and lipids also being synthesized rapidly from the primary photosynthetic products.

**Figure 1:**
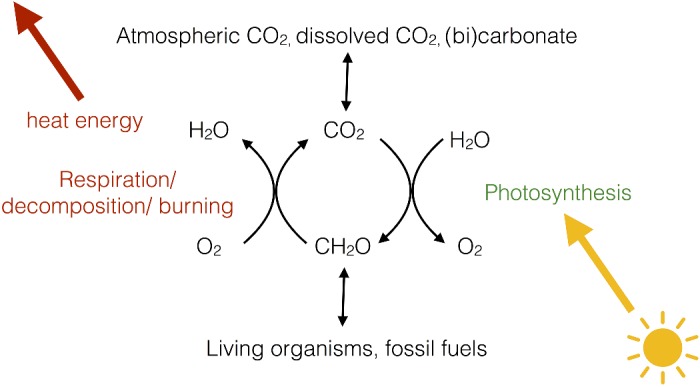
The global carbon cycle The relationship between respiration, photosynthesis and global CO_2_ and O_2_ levels.

The consideration of photosynthesis and respiration as opposing processes helps us to appreciate their role in shaping our environment. The fixation of CO_2_ by photosynthesis and its release during breakdown of organic molecules during respiration, decay and combustion of organic matter and fossil fuels can be visualized as the global carbon cycle (Figure 1).

At present, this cycle may be considered to be in a state of imbalance due to the burning of fossil fuels (fossilized photosynthesis), which is increasing the proportion of CO_2_ entering the Earth's atmosphere, leading to the so-called ‘greenhouse effect’ and human-made climate change.

Oxygenic photosynthesis is thought to have evolved only once during Earth's history in the cyanobacteria. All other organisms, such as plants, algae and diatoms, which perform oxygenic photosynthesis actually do so via cyanobacterial endosymbionts or ‘chloroplasts’. An endosymbiotoic event between an ancestral eukaryotic cell and a cyanobacterium that gave rise to plants is estimated to have occurred ∼1.5 billion years ago. Free-living cyanobacteria still exist today and are responsible for ∼50% of the world's photosynthesis. Cyanobacteria themselves are thought to have evolved from simpler photosynthetic bacteria that use either organic or inorganic compounds such a hydrogen sulfide as a source of electrons rather than water and thus do not produce oxygen.

### The site of photosynthesis in plants

**Figure 2:**
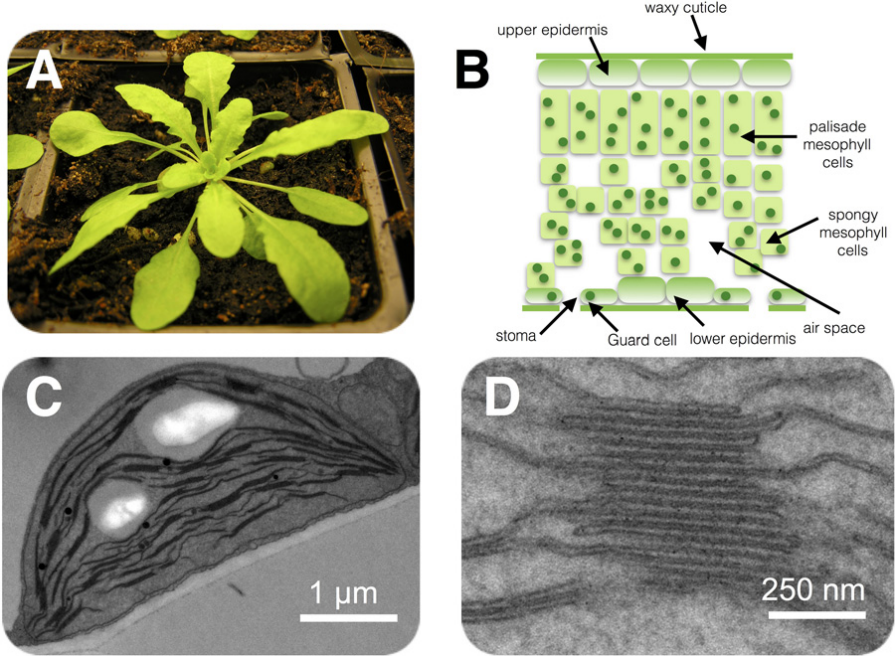
Location of the photosynthetic machinery (**A**) The model plant *Arabidopsis thaliana*. (**B**) Basic structure of a leaf shown in cross-section. Chloroplasts are shown as green dots within the cells. (**C**) An electron micrograph of an *Arabidopsis* chloroplast within the leaf. (**D**) Close-up region of the chloroplast showing the stacked structure of the thylakoid membrane.

In land plants, the principal organs of photosynthesis are the leaves (Figure 2A). Leaves have evolved to expose the largest possible area of green tissue to light and entry of CO_2_ to the leaf is controlled by small holes in the lower epidermis called stomata (Figure 2B). The size of the stomatal openings is variable and regulated by a pair of guard cells, which respond to the turgor pressure (water content) of the leaf, thus when the leaf is hydrated, the stomata can open to allow CO_2_ in. In contrast, when water is scarce, the guard cells lose turgor pressure and close, preventing the escape of water from the leaf via transpiration.

Within the green tissue of the leaf (mainly the mesophyll) each cell (∼100 μm in length) contains ∼100 chloroplasts (2–3 μm in length), the tiny organelles where photosynthesis takes place. The chloroplast has a complex structure (Figure 2C, D) with two outer membranes (the envelope), which are colourless and do not participate in photosynthesis, enclosing an aqueous space (the stroma) wherein sits a third membrane known as the thylakoid, which in turn encloses a single continuous aqueous space called the lumen.

**Figure 3:**
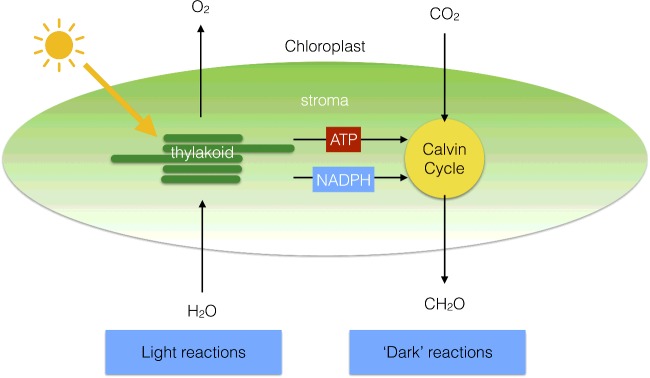
Division of labour within the chloroplast The light reactions of photosynthesis take place in the thylakoid membrane, whereas the dark reactions are located in the chloroplast stroma.

The light reactions of photosynthesis involve light-driven electron and proton transfers, which occur in the thylakoid membrane, whereas the dark reactions involve the fixation of CO_2_ into carbohydrate, via the Calvin–Benson cycle, which occurs in the stroma (Figure 3). The light reactions involve electron transfer from water to NADP^+^ to form NADPH and these reactions are coupled to proton transfers that lead to the phosphorylation of adenosine diphosphate (ADP) into ATP. The Calvin–Benson cycle uses ATP and NADPH to convert CO_2_ into carbohydrates (Figure 3), regenerating ADP and NADP^+^. The light and dark reactions are therefore mutually dependent on one another.

### Photosynthetic electron and proton transfer chain

**Figure 4:**
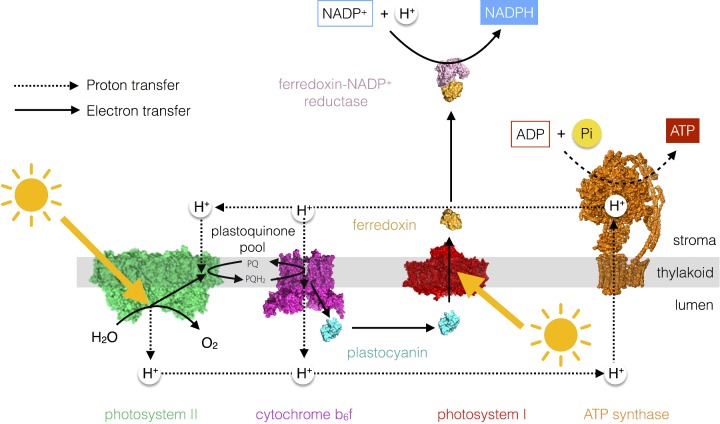
The photosynthetic electron and proton transfer chain The linear electron transfer pathway from water to NADP^+^ to form NADPH results in the formation of a proton gradient across the thylakoid membrane that is used by the ATP synthase enzyme to make ATP.

The light-driven electron transfer reactions of photosynthesis begin with the splitting of water by Photosystem II (PSII). PSII is a chlorophyll–protein complex embedded in the thylakoid membrane that uses light to oxidize water to oxygen and reduce the electron acceptor plastoquinone to plastoquinol. Plastoquinol in turn carries the electrons derived from water to another thylakoid-embedded protein complex called cytochrome *b*_6_*f* (cyt*b*_6_*f*). cyt*b*_6_*f* oxidizes plastoquinol to plastoquinone and reduces a small water-soluble electron carrier protein plastocyanin, which resides in the lumen. A second light-driven reaction is then carried out by another chlorophyll protein complex called Photosystem I (PSI). PSI oxidizes plastocyanin and reduces another soluble electron carrier protein ferredoxin that resides in the stroma. Ferredoxin can then be used by the ferredoxin–NADP^+^ reductase (FNR) enzyme to reduce NADP^+^ to NADPH. This scheme is known as the linear electron transfer pathway or Z-scheme (Figure 4).

**Figure 5:**
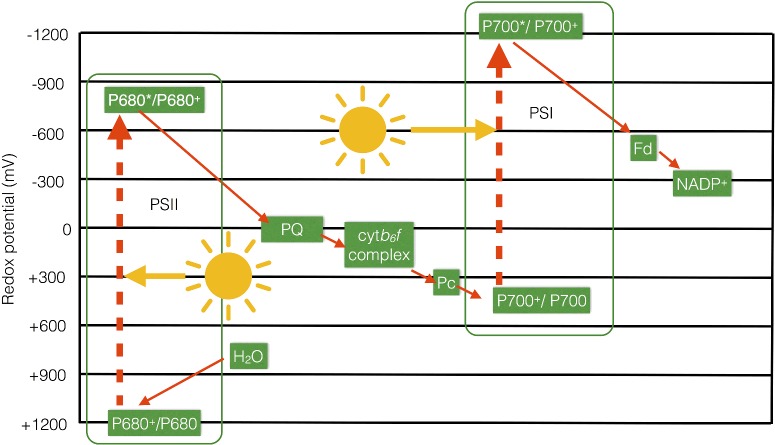
Z-scheme of photosynthetic electron transfer The main components of the linear electron transfer pathway are shown on a scale of redox potential to illustrate how two separate inputs of light energy at PSI and PSII result in the endergonic transfer of electrons from water to NADP^+^.

The Z-scheme, so-called since it resembles the letter ‘Z’ when turned on its side (Figure 5), thus shows how the electrons move from the water–oxygen couple (+820 mV) via a chain of redox carriers to NADP^+^/NADPH (−320 mV) during photosynthetic electron transfer. Generally, electrons are transferred from redox couples with low potentials (good reductants) to those with higher potentials (good oxidants) (e.g. during respiratory electron transfer in mitochondria) since this process is exergonic (see Box 2). However, photosynthetic electron transfer also involves two endergonic steps, which occur at PSII and at PSI and require an energy input in the form of light. The light energy is used to excite an electron within a chlorophyll molecule residing in PSII or PSI to a higher energy level; this excited chlorophyll is then able to reduce the subsequent acceptors in the chain. The oxidized chlorophyll is then reduced by water in the case of PSII and plastocyanin in the case of PSI.

The water-splitting reaction at PSII and plastoquinol oxidation at cyt*b*_6_*f* result in the release of protons into the lumen, resulting in a build-up of protons in this compartment relative to the stroma. The difference in the proton concentration between the two sides of the membrane is called a proton gradient. The proton gradient is a store of free energy (similar to a gradient of ions in a battery) that is utilized by a molecular mechanical motor ATP synthase, which resides in the thylakoid membrane (Figure 4). The ATP synthase allows the protons to move down their concentration gradient from the lumen (high H^+^ concentration) to the stroma (low H^+^ concentration). This exergonic reaction is used to power the endergonic synthesis of ATP from ADP and inorganic phosphate (P_i_). This process of photophosphorylation is thus essentially similar to oxidative phosphorylation, which occurs in the inner mitochondrial membrane during respiration.

An alternative electron transfer pathway exists in plants and algae, known as cyclic electron flow. Cyclic electron flow involves the recycling of electrons from ferredoxin to plastoquinone, with the result that there is no net production of NADPH; however, since protons are still transferred into the lumen by oxidation of plastoquinol by cyt*b*_6_*f*, ATP can still be formed. Thus photosynthetic organisms can control the ratio of NADPH/ATP to meet metabolic need by controlling the relative amounts of cyclic and linear electron transfer.

Box 2. **Relationship between redox potentials and standard free energy changes**

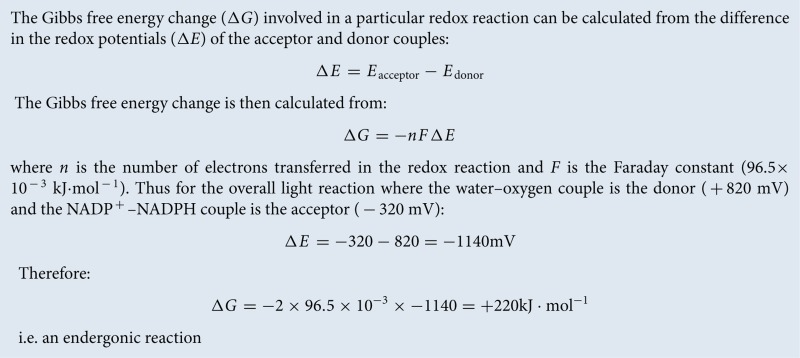

## How the photosystems work

### Light absorption by pigments

**Figure 6:**
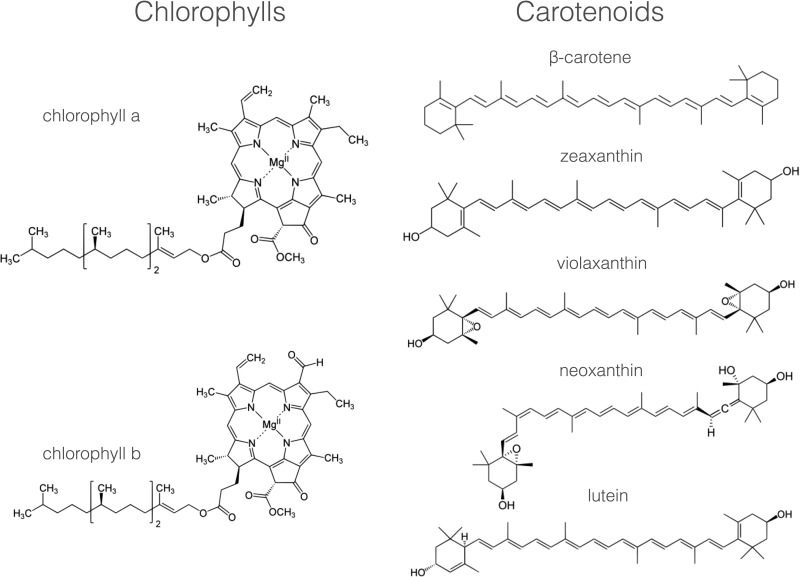
Major photosynthetic pigments in plants The chemical structures of the chlorophyll and carotenoid pigments present in the thylakoid membrane. Note the presence in each of a conjugated system of carbon–carbon double bonds that is responsible for light absorption.

Photosynthesis begins with the absorption of light by pigments molecules located in the thylakoid membrane. The most well-known of these is chlorophyll, but there are also carotenoids and, in cyanobacteria and some algae, bilins. These pigments all have in common within their chemical structures an alternating series of carbon single and double bonds, which form a conjugated system π–electron system (Figure 6).

**Figure 7:**
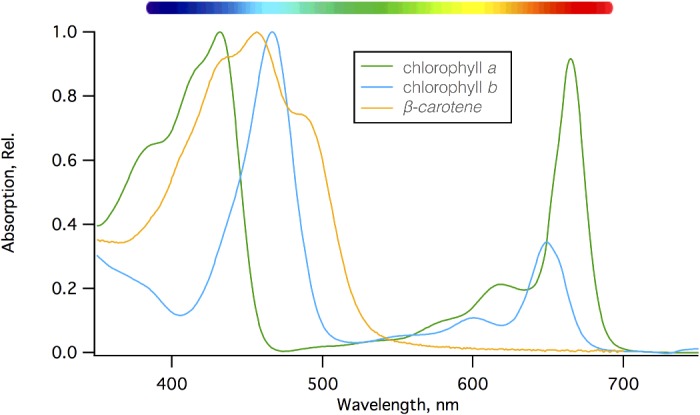
Basic absorption spectra of the major chlorophyll and carotenoid pigments found in plants Chlorophylls absorb light energy in the red and blue part of the visible spectrum, whereas carotenoids only absorb light in the blue/green.

The variety of pigments present within each type of photosynthetic organism reflects the light environment in which it lives; plants on land contain chlorophylls *a* and *b* and carotenoids such as β-carotene, lutein, zeaxanthin, violaxanthin, antheraxanthin and neoxanthin (Figure 6). The chlorophylls absorb blue and red light and so appear green in colour, whereas carotenoids absorb light only in the blue and so appear yellow/red (Figure 7), colours more obvious in the autumn as chlorophyll is the first pigment to be broken down in decaying leaves.

Light, or electromagnetic radiation, has the properties of both a wave and a stream of particles (light quanta). Each quantum of light contains a discrete amount of energy that can be calculated by multiplying Planck's constant, *h* (6.626×10^−34^ J·s) by ν, the frequency of the radiation in cycles per second (s^−1^):

E=hν

The frequency (ν) of the light and so its energy varies with its colour, thus blue photons (∼450 nm) are more energetic than red photons (∼650 nm). The frequency (ν) and wavelength (λ) of light are related by:

λ=c/ν

where *c* is the velocity of light (3.0×10^8^ m·s^−1^), and the energy of a particular wavelength (λ) of light is given by:

E=hc/λ

Thus 1 mol of 680 nm photons of red light has an energy of 176 kJ·mol^−1^.

**Figure 8:**
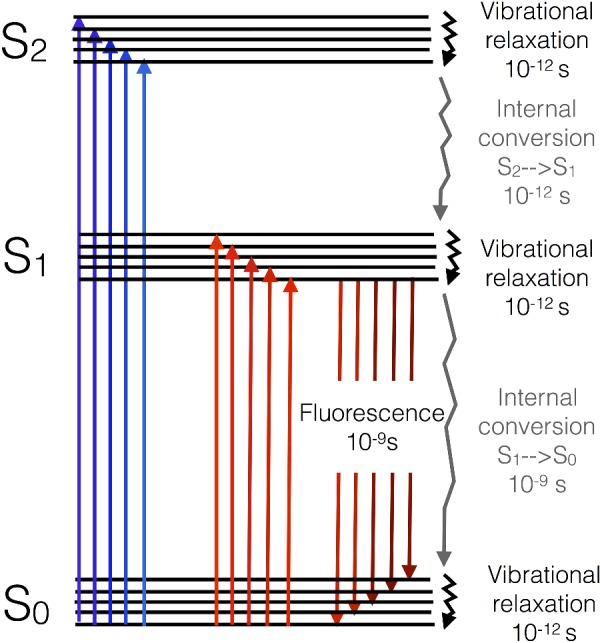
Jablonski diagram of chlorophyll showing the possible fates of the S_1_ and S_2_ excited states and timescales of the transitions involved Photons with slightly different energies (colours) excite each of the vibrational substates of each excited state (as shown by variation in the size and colour of the arrows).

The electrons within the delocalized π system of the pigment have the ability to jump up from the lowest occupied molecular orbital (ground state) to higher unoccupied molecular electron orbitals (excited states) via the absorption of specific wavelengths of light in the visible range (400–725 nm). Chlorophyll has two excited states known as S_1_ and S_2_ and, upon interaction of the molecule with a photon of light, one of its π electrons is promoted from the ground state (S_0_) to an excited state, a process taking just 10^−15^ s (Figure 8). The energy gap between the S_0_ and S_1_ states is spanned by the energy provided by a red photon (∼600–700 nm), whereas the energy gap between the S_0_ and S_2_ states is larger and therefore requires a more energetic (shorter wavelength, higher frequency) blue photon (∼400–500 nm) to span the energy gap.

**Figure 9:**
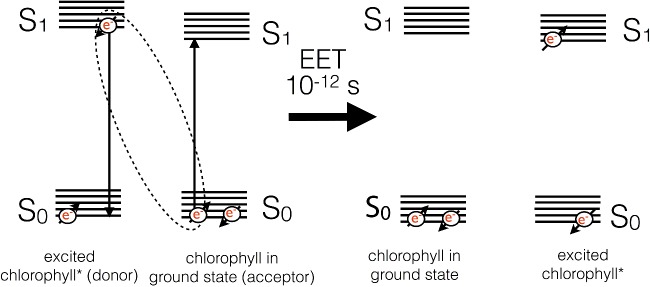
Basic mechanism of excitation energy transfer between chlorophyll molecules Two chlorophyll molecules with resonant S_1_ states undergo a mirror transition resulting in the non-radiative transfer of excitation energy between them.

Upon excitation, the electron in the S_2_ state quickly undergoes losses of energy as heat through molecular vibration and undergoes conversion into the energy of the S_1_ state by a process called internal conversion. Internal conversion occurs on a timescale of 10^−12^ s. The energy of a blue photon is thus rapidly degraded to that of a red photon. Excitation of the molecule with a red photon would lead to promotion of an electron to the S_1_ state directly. Once the electron resides in the S_1_ state, it is lower in energy and thus stable on a somewhat longer timescale (10^−9^ s). The energy of the excited electron in the S_1_ state can have one of several fates: it could return to the ground state (S_0_) by emission of the energy as a photon of light (fluorescence), or it could be lost as heat due to internal conversion between S_1_ and S_0_. Alternatively, if another chlorophyll is nearby, a process known as excitation energy transfer (EET) can result in the non-radiative exchange of energy between the two molecules (Figure 9). For this to occur, the two chlorophylls must be close by (<7 nm), have a specific orientation with respect to one another, and excited state energies that overlap (are resonant) with one another. If these conditions are met, the energy is exchanged, resulting in a mirror S_0_→S_1_ transition in the acceptor molecule and a S_1_→S_0_ transition in the other.

### Light-harvesting complexes

In photosynthetic systems, chlorophylls and carotenoids are found attached to membrane-embedded proteins known as light-harvesting complexes (LHCs). Through careful binding and orientation of the pigment molecules, absorbed energy can be transferred among them by EET. Each pigment is bound to the protein by a series of non-covalent bonding interactions (such as, hydrogen bonds, van der Waals interactions, hydrophobic interaction and co-ordination bonds between lone pair electrons of residues such as histidine in the protein and the Mg^2+^ ion in chlorophyll); the protein structure is such that each bound pigment experiences a slightly different environment in terms of the surrounding amino acid side chains, lipids, etc., meaning that the S_1_ and S_2_ energy levels are shifted in energy with respect to that of other neighbouring pigment molecules. The effect is to create a range of pigment energies that act to ‘funnel’ the energy on to the lowest-energy pigments in the LHC by EET.

### Reaction centres

**Figure 10:**
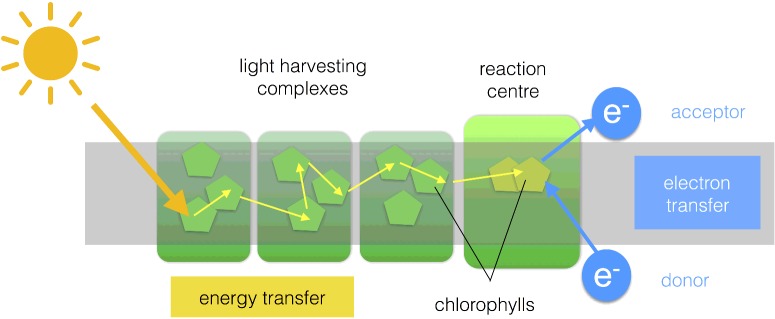
Basic structure of a photosystem Light energy is captured by the antenna pigments and transferred to the special pair of RC chlorophylls which undergo a redox reaction leading to reduction of an acceptor molecule. The oxidized special pair is regenerated by an electron donor.

A photosystem consists of numerous LHCs that form an antenna of hundreds of pigment molecules. The antenna pigments act to collect and concentrate excitation energy and transfer it towards a ‘special pair’ of chlorophyll molecules that reside in the reaction centre (RC) (Figure 10). Unlike the antenna pigments, the special pair of chlorophylls are ‘redox-active’ in the sense that they can return to the ground state (S_0_) by the transfer of the electron residing in the S_1_ excited state (Chl*) to another species. This process is known as charge separation and result in formation of an oxidized special pair (Chl^+^) and a reduced acceptor (A^−^). The acceptor in PSII is plastoquinone and in PSI it is ferredoxin. If the RC is to go on functioning, the electron deficiency on the special pair must be made good, in PSII the electron donor is water and in PSI it is plastocyanin.

It is worth asking why photosynthetic organisms bother to have a large antenna of pigments serving an RC rather than more numerous RCs. The answer lies in the fact that the special pair of chlorophylls alone have a rather small spatial and spectral cross-section, meaning that there is a limit to the amount of light they can efficiently absorb. The amount of light they can practically absorb is around two orders of magnitude smaller than their maximum possible turnover rate, Thus LHCs act to increase the spatial (hundreds of pigments) and spectral (several types of pigments with different light absorption characteristics) cross-section of the RC special pair ensuring that its turnover rate runs much closer to capacity.

### Photosystem II

**Figure 11:**
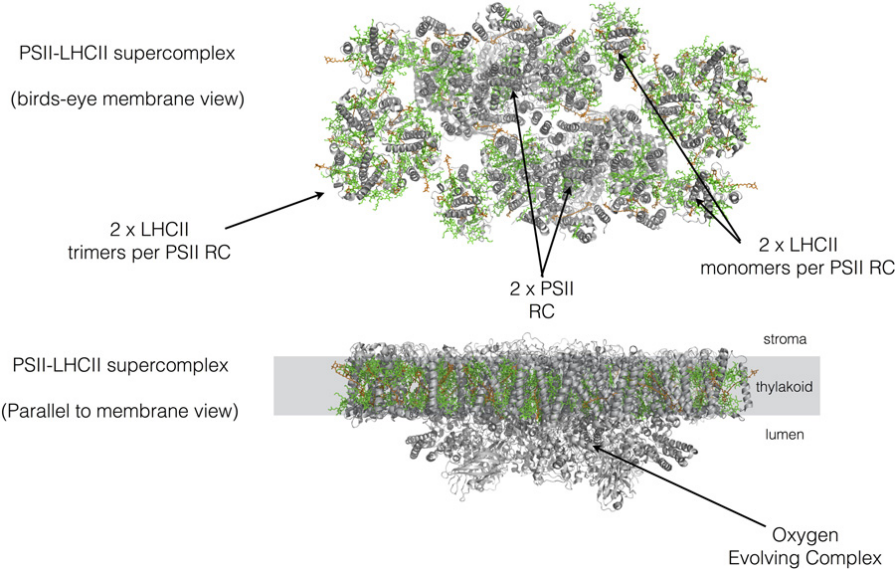
Basic structure of the PSII–LHCII supercomplex from spinach The organization of PSII and its light-harvesting antenna. Protein is shown in grey, with chlorophylls in green and carotenoids in orange. Drawn from PDB code 3JCU

**Figure 12:**
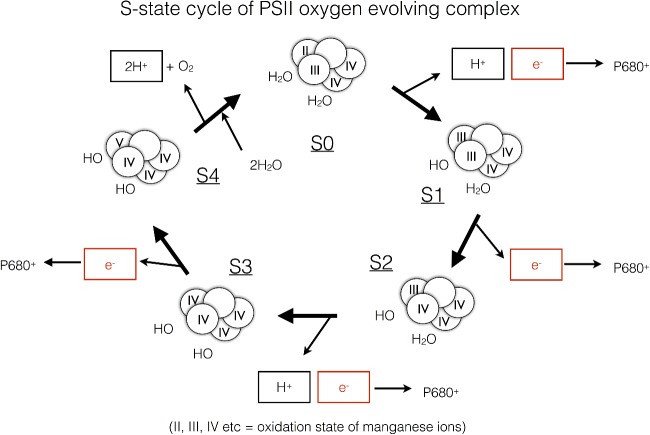
S-state cycle of water oxidation by the manganese cluster (shown as circles with roman numerals representing the manganese ion oxidation states) within the PSII oxygen-evolving complex Progressive extraction of electrons from the manganese cluster is driven by the oxidation of P680 within PSII by light. Each of the electrons given up by the cluster is eventually repaid at the S_4_ to S_0_ transition when molecular oxygen (O_2_) is formed. The protons extracted from water during the process are deposited into the lumen and contribute to the protonmotive force.

PSII is a light-driven water–plastoquinone oxidoreductase and is the only enzyme in Nature that is capable of performing the difficult chemistry of splitting water into protons, electrons and oxygen (Figure 11). In principle, water is an extremely poor electron donor since the redox potential of the water–oxygen couple is +820 mV. PSII uses light energy to excite a special pair of chlorophylls, known as P680 due to their 680 nm absorption peak in the red part of the spectrum. P680* undergoes charge separation that results in the formation of an extremely oxidizing species P680^+^ which has a redox potential of +1200 mV, sufficient to oxidize water. Nonetheless, since water splitting involves four electron chemistry and charge separation only involves transfer of one electron, four separate charge separations (turnovers of PSII) are required to drive formation of one molecule of O_2_ from two molecules of water. The initial electron donation to generate the P680 from P680^+^ is therefore provided by a cluster of manganese ions within the oxygen-evolving complex (OEC), which is attached to the lumen side of PSII (Figure 12). Manganese is a transition metal that can exist in a range of oxidation states from +1 to +5 and thus accumulates the positive charges derived from each light-driven turnover of P680. Progressive extraction of electrons from the manganese cluster is driven by the oxidation of P680 within PSII by light and is known as the S-state cycle (Figure 12). After the fourth turnover of P680, sufficient positive charge is built up in the manganese cluster to permit the splitting of water into electrons, which regenerate the original state of the manganese cluster, protons, which are released into the lumen and contribute to the proton gradient used for ATP synthesis, and the by-product O_2_. Thus charge separation at P680 provides the thermodynamic driving force, whereas the manganese cluster acts as a catalyst for the water-splitting reaction.

The electrons yielded by P680* following charge separation are not passed directly to plastoquinone, but rather via another acceptor called pheophytin, a porphyrin molecule lacking the central magnesium ion as in chlorophyll. Plastoquinone reduction to plastoquinol requires two electrons and thus two molecules of plastoquinol are formed per O_2_ molecule evolved by PSII. Two protons are also taken up upon formation of plastoquinol and these are derived from the stroma. PSII is found within the thylakoid membrane of plants as a dimeric RC complex surrounded by a peripheral antenna of six minor monomeric antenna LHC complexes and two to eight trimeric LHC complexes, which together form a PSII–LHCII supercomplex (Figure 11).

### Photosystem I

**Figure 13:**
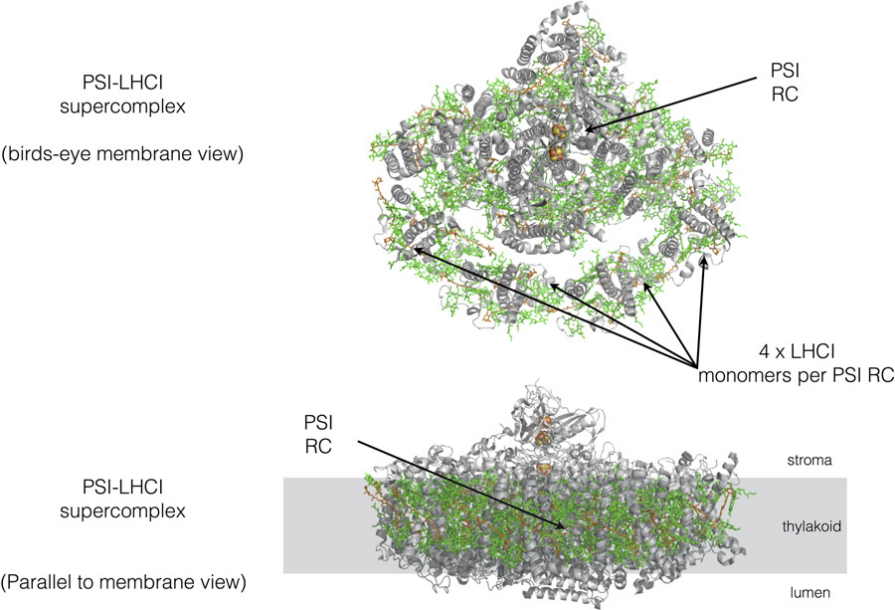
Basic structure of the PSI–LHCI supercomplex from pea The organization of PSI and its light-harvesting antenna. Protein is shown in grey, with chlorophylls in green and carotenoids in orange. Drawn from PDB code 4XK8.

PSI is a light-driven plastocyanin–ferredoxin oxidoreductase (Figure 13). In PSI, the special pair of chlorophylls are known as P700 due to their 700 nm absorption peak in the red part of the spectrum. P700* is an extremely strong reductant that is able to reduce ferredoxin which has a redox potential of −450 mV (and is thus is, in principle, a poor electron acceptor). Reduced ferredoxin is then used to generate NADPH for the Calvin–Benson cycle at a separate complex known as FNR. The electron from P700* is donated via another chlorophyll molecule and a bound quinone to a series of iron–sulfur clusters at the stromal side of the complex, whereupon the electron is donated to ferredoxin. The P700 species is regenerated form P700^+^ via donation of an electron from the soluble electron carrier protein plastocyanin.

PSI is found within the thylakoid membrane as a monomeric RC surrounded on one side by four LHC complexes known as LHCI. The PSI–LHCI supercomplex is found mainly in the unstacked regions of the thylakoid membrane (Figure 13).

## Other electron transfer chain components

### Plastoquinone/plastoquinol

Plastoquinone is a small lipophilic electron carrier molecule that resides within the thylakoid membrane and carries two electrons and two protons from PSII to the cyt*b*_6_*f* complex. It has a very similar structure to that of the molecule ubiquinone (coenzyme Q_10_) in the mitochondrial inner membrane.

### Cytochrome *b_6_f* complex

**Figure 14:**
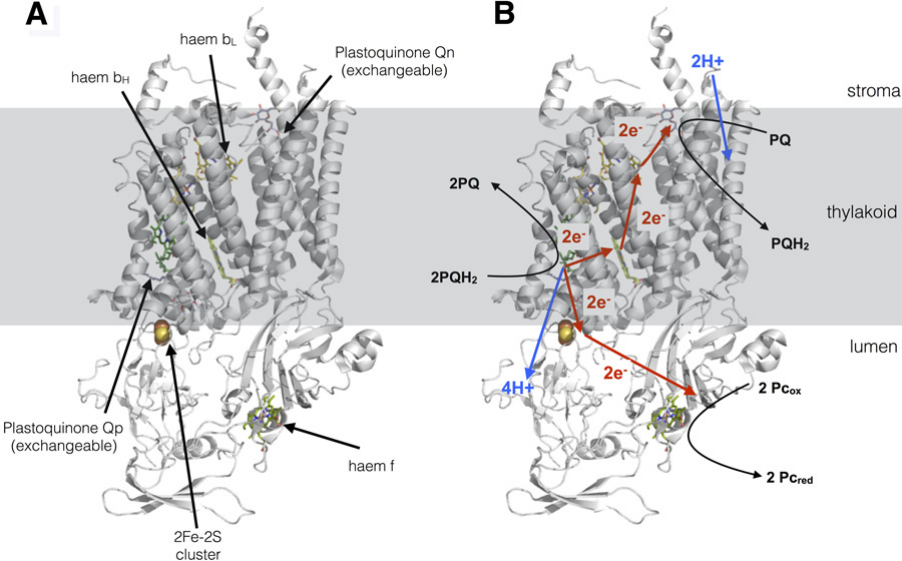
Cytochrome *b*_6_*f* complex (**A**) Structure drawn from PDB code 1Q90. (**B**) The protonmotive Q-cycle showing how electrons from plastoquinol are passed to both plastocyanin and plastoquinone, doubling the protons deposited in the lumen for every plastoquinol molecule oxidized by the complex.

The cyt*b*_6_*f* complex is a plastoquinol–plastocyanin oxidoreductase and possess a similar structure to that of the cytochrome *bc*_1_ complex (complex III) in mitochondria (Figure 14A). As with Complex III, cyt*b_6_f* exists as a dimer in the membrane and carries out both the oxidation and reduction of quinones via the so-called Q-cycle. The Q-cycle (Figure 14B) involves oxidation of one plastoquinol molecule at the Qp site of the complex, both protons from this molecule are deposited in the lumen and contribute to the proton gradient for ATP synthesis. The two electrons, however, have different fates. The first is transferred via an iron–sulfur cluster and a haem cofactor to the soluble electron carrier plastocyanin (see below). The second electron derived from plastoquinol is passed via two separate haem cofactors to another molecule of plastoquinone bound to a separate site (Qn) on the complex, thus reducing it to a semiquinone. When a second plastoquinol molecule is oxidized at Qp, a second molecule of plastocyanin is reduced and two further protons are deposited in the lumen. The second electron reduces the semiquinone at the Qn site which, concomitant with uptake of two protons from the stroma, causes its reduction to plastoquinol. Thus for each pair of plastoquinol molecules oxidized by the complex, one is regenerated, yet all four protons are deposited into the lumen. The Q-cycle thus doubles the number of protons transferred from the stroma to the lumen per plastoquinol molecule oxidized.

### Plastocyanin

Plastocyanin is a small soluble electron carrier protein that resides in the thylakoid lumen. The active site of the plastocyanin protein binds a copper ion, which cycles between the Cu^2+^ and Cu^+^ oxidation states following its oxidation by PSI and reduction by cyt*b*_6_*f* respectively.

### Ferredoxin

Ferredoxin is a small soluble electron carrier protein that resides in the chloroplast stroma. The active site of the ferredoxin protein binds an iron–sulfur cluster, which cycles between the Fe^2+^ and Fe^3+^ oxidation states following its reduction by PSI and oxidation by the FNR complex respectively.

### Ferredoxin–NADP^+^ reductase

The FNR complex is found in both soluble and thylakoid membrane-bound forms. The complex binds a flavin–adenine dinucleotide (FAD) cofactor at its active site, which accepts two electrons from two molecules of ferredoxin before using them reduce NADP^+^ to NADPH.

### ATP synthase

The ATP synthase enzyme is responsible for making ATP from ADP and P_i_; this endergonic reaction is powered by the energy contained within the protonmotive force. According to the structure, 4.67 H^+^ are required for every ATP molecule synthesized by the chloroplast ATP synthase. The enzyme is a rotary motor which contains two domains: the membrane-spanning F_O_ portion which conducts protons from the lumen to the stroma, and the F_1_ catalytic domain that couples this exergonic proton movement to ATP synthesis.

### Membrane stacking and the regulation of photosynthesis

**Figure 15:**
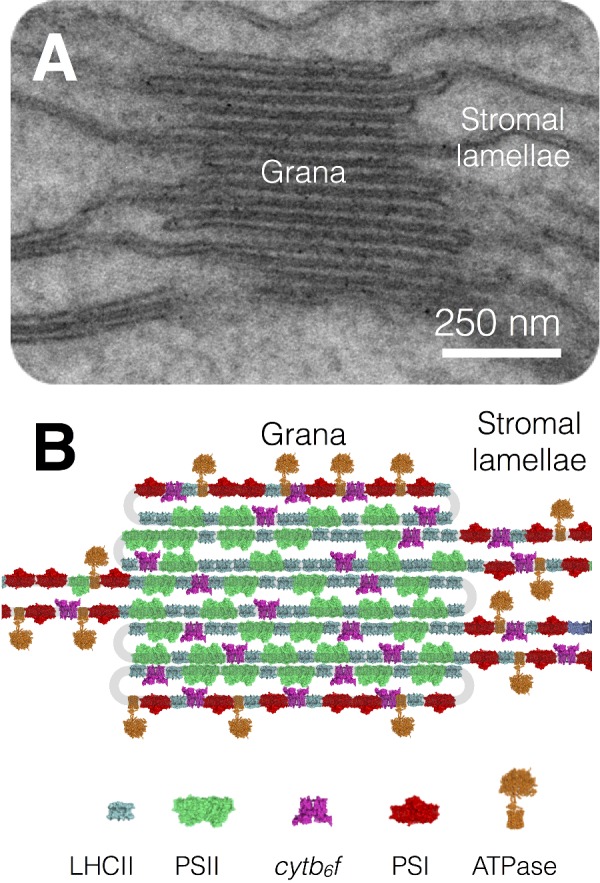
Lateral heterogeneity in thylakoid membrane organization (**A**) Electron micrograph of the thylakoid membrane showing stacked grana and unstacked stromal lamellae regions. (**B**) Model showing the distribution of the major complexes of photosynthetic electron and proton transfer between the stacked grana and unstacked stromal lamellae regions.

Within the thylakoid membrane, PSII–LHCII supercomplexes are packed together into domains known as the grana, which associate with one another to form grana stacks. PSI and ATP synthase are excluded from these stacked PSII–LHCII regions by steric constraints and thus PSII and PSI are segregated in the thylakoid membrane between the stacked and unstacked regions (Figure 15). The cyt*b*_6_*f* complex, in contrast, is evenly distributed throughout the grana and stromal lamellae. The evolutionary advantage of membrane stacking is believed to be a higher efficiency of electron transport by preventing the fast energy trap PSI from ‘stealing’ excitation energy from the slower trap PSII, a phenomenon known as spillover. Another possible advantage of membrane stacking in thylakoids may be the segregation of the linear and cyclic electron transfer pathways, which might otherwise compete to reduce plastoquinone. In this view, PSII, cyt*b*_6_*f* and a sub-fraction of PSI closest to the grana is involved in linear flow, whereas PSI and cyt*b*_6_*f* in the stromal lamellae participates in cyclic flow. The cyclic electron transfer pathway recycles electrons from ferredoxin back to plastoquinone and thus allows protonmotive force generation (and ATP synthesis) without net NADPH production. Cyclic electron transfer thereby provides the additional ATP required for the Calvin–Benson cycle (see below).

### ‘Dark’ reactions: the Calvin–Benson cycle

**Figure 16:**
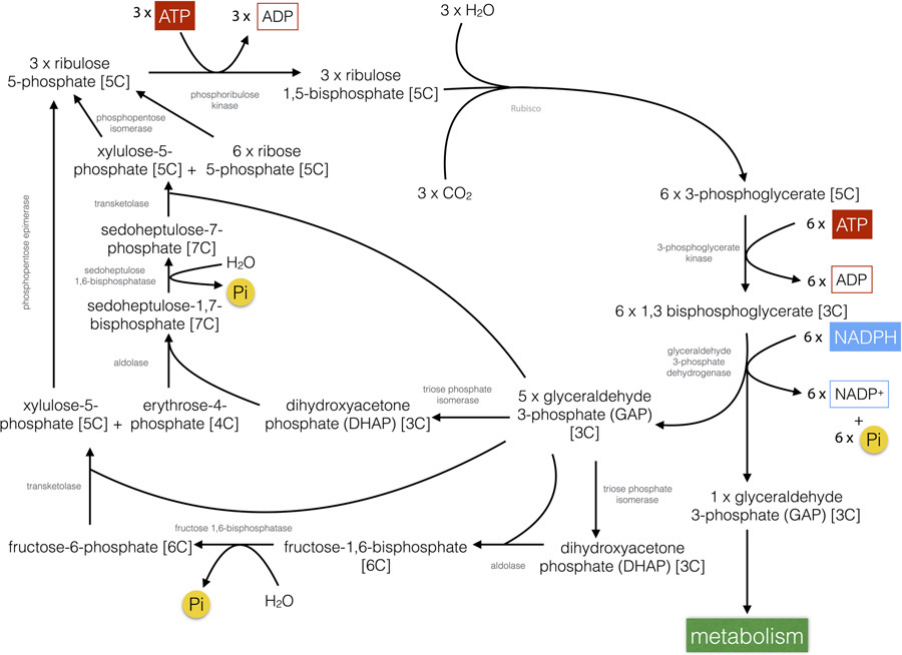
The Calvin–Benson cycle Overview of the biochemical pathway for the fixation of CO_2_ into carbohydrate in plants.

CO_2_ is fixed into carbohydrate via the Calvin–Benson cycle in plants, which consumes the ATP and NADPH produced during the light reactions and thus in turn regenerates ADP, P_i_ and NADP^+^. In the first step of the Calvin–Benson cycle (Figure 16), CO_2_ is combined with a 5-carbon (5C) sugar, ribulose 1,5-bisphosphate in a reaction catalysed by the enzyme ribulose-1,5-bisphosphate carboxylase/oxygenase (Rubisco). The reaction forms an unstable 6C intermediate that immediately splits into two molecules of 3-phosphoglycerate. 3-Phosphoglycerate is first phosphorylated by 3-phosphoglycerate kinase using ATP to form 1,3-bisphosphoglycerate. 1,3-Bisphosphoglycerate is then reduced by glyceraldehyde 3-phosphate dehydrogenase using NADPH to form glyceraldehyde 3-phosphate (GAP, a triose or 3C sugar) in reactions, which are the reverse of glycolysis. For every three CO_2_ molecules initially combined with ribulose 1,5-bisphopshate, six molecules of GAP are produced by the subsequent steps. However only one of these six molecules can be considered as a product of the Calvin–Benson cycle since the remaining five are required to regenerate ribulose 1,5-bisphosphate in a complex series of reactions that also require ATP. The one molecule of GAP that is produced for each turn of the cycle can be quickly converted by a range of metabolic pathways into amino acids, lipids or sugars such as glucose. Glucose in turn may be stored as the polymer starch as large granules within chloroplasts.

A complex biochemical ‘dance’ (Figure 16) is then involved in the regeneration of three ribulose 1,5-bisphosphate (5C) from the remaining five GAP (3C) molecules. The regeneration begins with the conversion of two molecules of GAP into dihydroxyacetone phosphate (DHAP) by triose phosphate isomerase; one of the DHAP molecules is the combined with another GAP molecule to make fructose 1,6-bisphosphate (6C) by aldolase. The fructose 1,6-bisphosphate is then dephosphorylated by fructose-1,6-bisphosphatase to yield fructose 6-phosphate (6C) and releasing P_i_. Two carbons are then removed from fructose 6-phosphate by transketolase, generating erythrose 4-phosphate (4C); the two carbons are transferred to another molecule of GAP generating xylulose 5-phosphate (5C). Another DHAP molecule, formed from GAP by triose phosphate isomerase is then combined with the erythrose 4-phosphate by aldolase to form sedoheptulose 1,7-bisphosphate (7C). Sedoheptulose 1,7-bisphosphate is then dephosphorylated to sedoheptulose 7-phosphate (7C) by sedoheptulose-1,7-bisphosphatase releasing P_i_. Sedoheptulose 7-phosphate has two carbons removed by transketolase to produce ribose 5-phosphate (5C) and the two carbons are transferred to another GAP molecule producing another xylulose 5-phosphate (5C). Ribose 5-phosphate and the two molecules of xylulose 5-phosphate (5C) are then converted by phosphopentose isomerase to three molecules of ribulose 5-phosphate (5C). The three ribulose 5-phosphate molecules are then phosphorylated using three ATP by phosphoribulokinase to regenerate three ribulose 1,5-bisphosphate (5C).

Overall the synthesis of 1 mol of GAP requires 9 mol of ATP and 6 mol of NADPH, a required ratio of 1.5 ATP/NADPH. Linear electron transfer is generally thought to supply ATP/NADPH in a ratio of 1.28 (assuming an H^+^/ATP ratio of 4.67) with the shortfall of ATP believed to be provided by cyclic electron transfer reactions. Since the product of the Calvin cycle is GAP (a 3C sugar) the pathway is often referred to as C_3_ photosynthesis and plants that utilize it are called C_3_ plants and include many of the world's major crops such as rice, wheat and potato.

Many of the enzymes involved in the Calvin–Benson cycle (e.g. transketolase, glyceraldehyde-3-phosphate dehydrogenase and aldolase) are also involved in the glycolysis pathway of carbohydrate degradation and their activity must therefore be carefully regulated to avoid futile cycling when light is present, i.e. the unwanted degradation of carbohydrate. The regulation of the Calvin–Benson cycle enzymes is achieved by the activity of the light reactions, which modify the environment of the dark reactions (i.e. the stroma). Proton gradient formation across the thylakoid membrane during the light reactions increases the pH and also increases the Mg^2+^ concentration in the stroma (as Mg^2+^ flows out of the lumen as H^+^ flows in to compensate for the influx of positive charges). In addition, by reducing ferredoxin and NADP^+^, PSI changes the redox state of the stroma, which is sensed by the regulatory protein thioredoxin. Thioredoxin, pH and Mg^2+^ concentration play a key role in regulating the activity of the Calvin–Benson cycle enzymes, ensuring the activity of the light and dark reactions is closely co-ordinated.

### Rubisco

**Figure 17:**
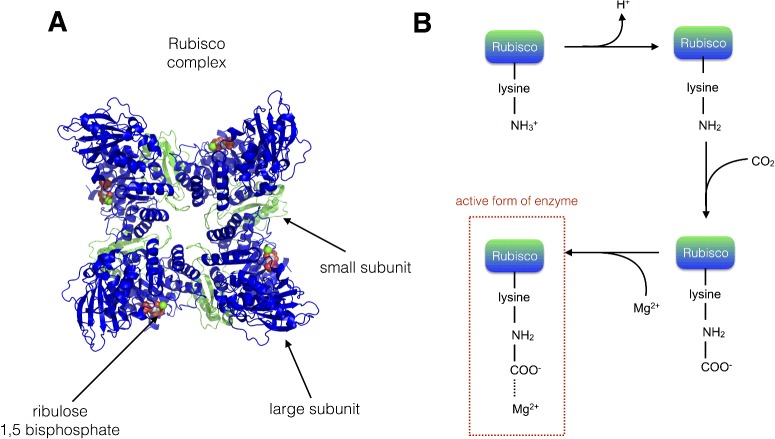
Rubisco (**A**) Structure of the Rubisco enzyme (the large subunits are shown in blue and the small subunits in green); four of each type of subunit are visible in the image. Drawn from PDB code 1RXO. (**B**) Activation of the lysine residue within the active site of Rubisco occurs via elevated stromal pH and Mg^2+^ concentration as a result of the activity of the light reactions.

It is noteworthy that, despite the complexity of the dark reactions outlined above, the carbon fixation step itself (i.e. the incorporation of CO_2_ into carbohydrate) is carried out by a single enzyme, Rubisco. Rubisco is a large multisubunit soluble protein complex found in the chloroplast stroma. The complex consists of eight large (56 kDa) subunits, which contain both catalytic and regulatory domains, and eight small subunits (14 kDa), which enhance the catalytic function of the L subunits (Figure 17A). The carboxylation reaction carried out by Rubisco is highly exergonic (Δ*G*°=−51.9 kJ·mol-^1^), yet kinetically very slow (just 3 s^−1^) and begins with the protonation of ribulose 1,5-bisphosphate to form an enediolate intermediate which can be combined with CO_2_ to form an unstable 6C intermediate that is quickly hydrolysed to yield two 3C 3-phosphoglycerate molecules. The active site in the Rubisco enzyme contains a key lysine residue, which reacts with another (non-substrate) molecule of CO_2_ to form a carbamate anion that is then able to bind Mg^2+^. The Mg^2+^ in the active site is essential for the catalytic function of Rubisco, playing a key role in binding ribulose 1,5-bisphosphate and activating it such that it readily reacts with CO_2.._ Rubisco activity is co-ordinated with that of the light reactions since carbamate formation requires both high Mg^2+^ concentration and alkaline conditions, which are provided by the light-driven changes in the stromal environment discussed above (Figure 17B).

In addition to carboxylation, Rubisco also catalyses a competitive oxygenation reaction, known as photorespiration, that results in the combination of ribulose 1,5-bisphosphate with O_2_ rather than CO_2_. In the oxygenation reaction, one rather than two molecules of 3-phosphoglycerate and one molecule of a 2C sugar known as phosphoglycolate are produced by Rubisco. The phosphoglycolate must be converted in a series of reactions that regenerate one molecule of 3-phosphoglycerate and one molecule of CO_2_. These reactions consume additional ATP and thus result in an energy loss to the plant. Although the oxygenation reaction of Rubisco is much less favourable than the carboxylation reaction, the relatively high concentration of O_2_ in the leaf (250 μM) compared with CO_2_ (10 μM) means that a significant amount of photorespiration is always occurring. Under normal conditions, the ratio of carboxylation to oxygenation is between 3:1 and 4:1. However, this ratio can be decreased with increasing temperature due to decreased CO_2_ concentration in the leaf, a decrease in the affinity of Rubisco for CO_2_ compared with O_2_ and an increase in the maximum rate of the oxygenation reaction compared with the carboxylation reaction. The inefficiencies of the Rubisco enzyme mean that plants must produce it in very large amounts (∼30–50% of total soluble protein in a spinach leaf) to achieve the maximal photosynthetic rate.

### CO_2_-concentrating mechanisms

**Figure 18:**
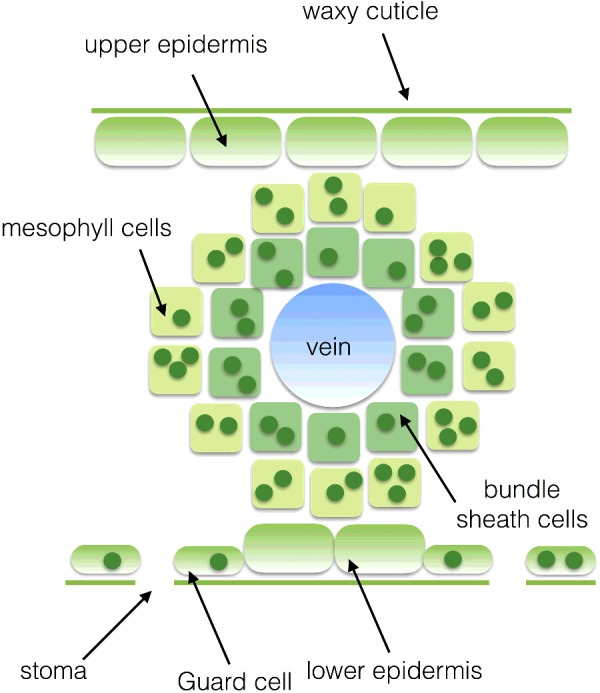
Diagram of a C_4_ plant leaf showing Kranz anatomy

**Figure 19:**
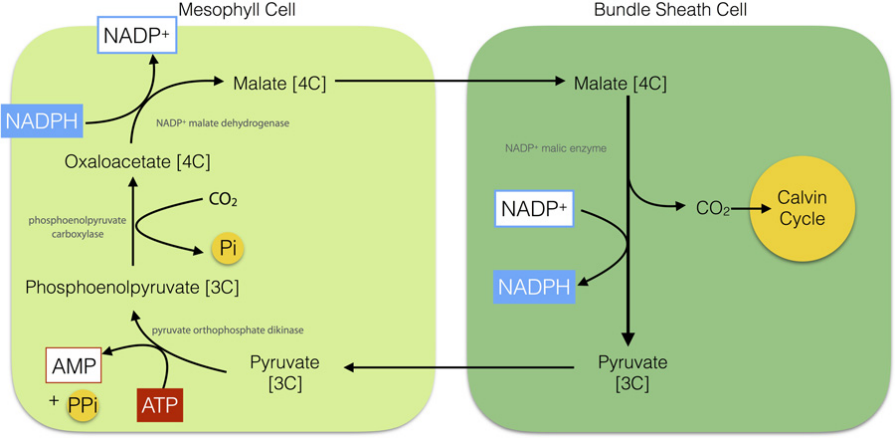
The C_4_ pathway (NADP^+^–malic enzyme type) for fixation of CO_2_

To counter photorespiration, plants, algae and cyanobacteria have evolved different CO_2_-concentrating mechanisms CCMs that aim to increase the concentration of CO_2_ relative to O_2_ in the vicinity of Rubisco. One such CCM is C_4_ photosynthesis that is found in plants such as maize, sugar cane and savanna grasses. C_4_ plants show a specialized leaf anatomy: Kranz anatomy (Figure 18). Kranz, German for wreath, refers to a bundle sheath of cells that surrounds the central vein within the leaf, which in turn are surrounded by the mesophyll cells. The mesophyll cells in such leaves are rich in the enzyme phosphoenolpyruvate (PEP) carboxylase, which fixes CO_2_ into a 4C carboxylic acid: oxaloaceatate. The oxaloacetate formed by the mesophyll cells is reduced using NADPH to malate, another 4C acid: malate. The malate is then exported from the mesophyll cells to the bundle sheath cells, where it is decarboxylated to pyruvate thus regenerating NADPH and CO_2_. The CO_2_ is then utilized by Rubisco in the Calvin cycle. The pyruvate is in turn returned to the mesophyll cells where it is phosphorylated using ATP to reform PEP (Figure 19). The advantage of C_4_ photosynthesis is that CO_2_ accumulates at a very high concentration in the bundle sheath cells that is then sufficient to allow Rubisco to operate efficiently.

Plants growing in hot, bright and dry conditions inevitably have to have their stomata closed for large parts of the day to avoid excessive water loss and wilting. The net result is that the internal CO_2_ concentration in the leaf is very low, meaning that C_3_ photosynthesis is not possible. To counter this limitation, another CCM is found in succulent plants such as cacti. The Crassulaceae fix CO_2_ into malate during the day via PEP carboxylase, store it within the vacuole of the plant cell at night and then release it within their tissues by day to be fixed via normal C_3_ photosynthesis. This is termed crassulacean acid metabolism (CAM).

## Notes

This article is a reviewed, revised and updated version of the following ‘Biochemistry Across the School Curriculum’ (BASC) booklet: Weaire, P.J. (1994) *Photosynthesis*. For further information and to provide feedback on this or any other Biochemical Society education resource, please contact education@biochemistry.org. For further information on other Biochemical Society publications, please visit www.biochemistry.org/publications.

## Abbreviations

ADP: adenosine diphosphate
ATP: adenosine triphosphate
CH_2_O: carbohydrate
cyt*b*_6_*f*: cytochrome *b*_6_*f*
DHAP: dihydroxyacetone phosphate
EET: excitation energy transfer
FNR: ferredoxin–NADP^+^ reductase
GAP: glyceraldehyde 3-phosphate
LHC: light-harvesting complex
NADPH: nicotinomide–adenine dinucleotide phosphate
PEP: phosphoenolpyruvate
P_i_: inorganic phosphate
PSI: Photosystem I
PSII: Photosystem II
RC: reaction centre
Rubisco: ribulose-1,5-bisphosphate carboxylase/oxygenase
