# Correction: Photosynthesis

**DOI:** 10.1042/EBC20160016_COR

**Published:** 2017-10-31

**Authors:** Matthew P. Johnson

**Essays Biochem. (2016) volume 60, issue 3, pages 255–273;** **https://doi.org/10.1042/EBC20160016**

The penultimate sentence of article was factually incorrect as published; the corrected sentence is presented here (corrections underlined):

“The Crassulaceae fix CO_2_ into malate during the night via PEP carboxylase, store it within the vacuole of the plant cell at night and in the daytime the malate is transported to chloroplasts to be fixed via normal C3 photosynthesis.”

